# Investigation into the Effect of Spinel Pigments on the Photostability and Combustion Properties of Ethylene-Norbornene Copolymer

**DOI:** 10.3390/ma14144050

**Published:** 2021-07-20

**Authors:** Małgorzata Kuśmierek, Bolesław Szadkowski, Przemysław Rybiński, Magdalena Śliwka-Kaszyńska, Mirosława Prochoń, Bartłomiej Syrek, Anna Marzec

**Affiliations:** 1 Institute of Polymer and Dye Technology, Faculty of Chemistry, Lodz University of Technology, Stefanowskiego 12/16, 90-924 Lodz, Poland; malgorzata.kusmierek@dokt.p.lodz.pl (M.K.); boleslaw.szadkowski@dokt.p.lodz.pl (B.S.); miroslawa.prochon@p.lodz.pl (M.P.); 2 Institute of Chemistry, The Jan Kochanowski University, Uniwersytecka 7, 25-369 Kielce, Poland; bartlomiejsyrek@wp.pl; 3 Department of Organic Chemistry, Faculty of Chemistry, Gdańsk University of Technology, 80-233 Gdańsk, Poland; magdalena.sliwka-kaszynska@pg.edu.pl

**Keywords:** polymer composites, coloring agent, spinel pigment, composite properties, UV aging

## Abstract

Multicolor ethylene-norbornene (EN) composites filled with three different spinel pigments (Cobalt Green-PG50, Zinc Iron Yellow-PY 119, Praseodym Yellow-PY159) were prepared by melt mixing and characterized in terms of their stability under destructive environmental conditions. The EN films were subjected to accelerated aging by ultraviolet (UV) photooxidation for 300 h, 600 h, or 900 h. The mechanical performance of the EN composites was investigated in static and dynamic mechanical tests. The morphologies of the EN samples and their color changes during the aging process were evaluated by scanning electron microscopy (SEM) and spectrophotometric measurements. Fourier transform infrared (FTIR) spectroscopy was applied to determine the amount of carbonyl groups resulting from surface oxidation at different aging times. The effects of the spinel pigments on the thermal stability and combustion properties of the multicolor polymer composites were also assessed, and compared with a sample containing the organic Pigment Yellow 139 (PY139). The results show that the color changes (ΔE) in the spinel pigments were minor in comparison to those in the organic pigment (PY139) and the reference film. The Zinc Yellow (PY119) pigment was the most effective stabilizer of EN copolymer. Moreover, the spinel pigments had a positive effect on the flame retardancy of the EN composites. Microcombustion tests (MCC) showed that the incorporation of both the spinels and the organic pigment PY139 into the EN matrix reduced the heat release rate (HRR) and total heat release (THR) parameters.

## 1. Introduction

Organic and inorganic pigments are an important group of additives. They not only give color to the materials to which they are added, but can also improve their applicative properties, such as light stability and flammability [1,2,3,4]. Pigments can be categorized based on their origin (natural or synthetic) and chemical composition (organic or inorganic) [5]. Common classes of organic pigments used in polymers include phthalocyanines, isoindolinones, perylenes, anthraquinones, flavanthrones, thioindigos, quinophthalones, azocondensations, and azomethine metal complexes. Inorganic pigments fall into different classes, according to their chemical content, such as oxides, chromates, sulfates, silicates, borates, molybdates, phosphates, vanadates, iron cyanate, hydroxides, sulfides, and metals [6,7]. Organic pigments are brightly colored, but generally not as lightfast or opaque as inorganic pigments. Due to their thermal, chemical, and solar instability, organic coloring materials are restricted to short-term applications and are not suitable for ceramic and polymer processing at high temperatures [8].

Spinel pigments are mixed metal oxides with the general formula AB_2_O_4_. They are stable toward sunlight, heat, and environmental changes [9]. The A^2+^B^3+^_2_O_4_ spinel system consists of divalent (^2+^) and trivalent (^3+^) metal ions in one of two distinct coordination environments. Another type of spinel pigment is the B[AB]O_4_ spinel system. Known as “inverse spinels”, the A represents metal ions situated in octahedral structures, whereas B ions appear in tetrahedral and octahedral sites [10]. Spinel pigments have found widespread applications as catalysts, semiconductors, and magnetic ceramic powders. As colorants, these pigments are most often used in ceramics, due to their chemical and thermal stability [11,12].

Recent research in pigmentation technology has been aimed at lowering energy costs related to cooling by providing infrared absorbance or reflectance. Colorants in paints and coatings can reflect invisible heat, especially from solar radiation [13,14,15,16,17]. Approximately 40% of solar radiation consists of the near-infrared (NIR) radiation wavelength range from 780 nm to 1000 nm [18,19]. Therefore, painting or coating objects with NIR reflective additives can reduce heating, which is beneficial in automotive and civil engineering applications, among others [20,21,22,23]. Cool pigments commonly used in urban engineering are light-colored or white. However, darker and more vivid colorants are desirable for aesthetic reasons. Spinel pigments are available in a wide range of colors, and exhibit infrared reflectance properties [24,25]. Spinel pigments are currently used in acrylic and alkyd resins, to obtain paints, inks, and coatings [26,27]. Their high thermal stability, broad range of colors, and promising UV-Vis and NIR reflectance make them promising candidates for use in high-performance polymer composites.

The aging behavior of polymers, blends, and composites under different environmental conditions is a crucial consideration when assessing the possible applications of additives and establishing the lifetime of the final products.

Organic and inorganic pigments can have either favorable or unfavorable effects on the properties of polymer composites, such as thermal or light stability [28]. Different properties of pigments, such as light absorbing characteristics or photochemical behavior, influence polymer materials during aging, especially under UV light irradiation. As a consequence, pigments can provide stability by absorbing and/or scattering harmful radiation or contribute to sensitize the degradation of the polymer materials, for example by generating singlet oxygen or via hydrogen abstraction by photoexcited pigment molecules [29]. Uzelmeier [30] observed that organic pigments such as phthalocyanine blue and green, or quinacridone magenta, and inorganic pigments including carbon black, cadmium yellow, mercadmium red, and ultramarine blue, enhanced both the thermal stability and photostability of polypropylene materials. A study on cadmium yellow, ultramarine blue, phthalocyanine green, and blue pigments showed that all colorants contributed to improve the stability of LDPE films [31]. Steinlin and Saar [32] investigated the impact of pigments on the light stability of polypropylene (PP) fiber additionally containing the commercial light stabilizer Tinuvin 770. They concluded that a significant number of the pigments had a negative effect on the light stability of the PP fibers, especially the yellow, red, and orange colorants (e.g., P. Yellow 94, P. Yellow 83, P. Red 224, P. Yellow 109, P. Orange 31, P. Yellow 110). Green, blue, and black pigments (P. Black 7, P. Blue 60, P. Violet 37, P. Blue 16, P. Red 177, P. Red 220, P. Blue 15:3, P. Green 7) improved the light stability of the fiber, despite the presence of the Tinuvin 770 stabilizer.

Carbon black and phthalocyanine pigments induce a marked protective effect in many polymers. Gilroy and Chan [33] noticed that some organic pigments (e.g., P. Blue 15, P. Red 220, P. Green 7, P. Green 36, P. Yellow 14) produced a favorable effect on the thermal stability polyolefin wires and cables. Black, brown, and red pigments were recommended to improve the color and physical properties of ABS under weather aging. In rigid PVC, most of the pigments contributed to improved light stability under exposure to outdoor conditions. The surfaces degraded much faster than the bulk of the polymer, with only minor differences in surface protection by the various organic and inorganic pigments (phthalocyanine blue, iron oxide red, channel black, P. Red 48, P. Yellow 83) [34].

Accelerated degradation tests are often used to simulate aging processes and select appropriate additives and stabilizers. There are no studies in the literature describing the impact of spinel colorants on the properties of polymers during weathering. In our previous work, we studied the impact of earth pigments on the aging process and combustion behavior of ethylene-norbornene copolymers [35]. By adding earth pigments, we obtained materials with attractive colors and improved resistance to unfavorable outdoor conditions. In the present research, we prepared thermoplastic composites using ethylene-norbornene copolymer with selected spinel colorants: Cobalt Green, Zinc Iron Yellow, and Praseodym Yellow powders. The mechanical, colorimetric, and surface changes were monitored under UV exposure (300 h, 600 h, 900 h). The effect of the colorants on the combustion properties and thermal stability of the ethylene-norbornene copolymers was studied using microscale combustion calorimetry and thermal analysis. We compared the behavior during aging of the polymers containing spinels to an EN film containing organic pigment PY139.

## 2. Materials and Methods

### 2.1. Materials

Ethylene-norbornene (EN) random copolymer with the commercial name Topas Elastomer 140 was provided by TOPAS Advanced Polymers (Germany). Three spinel pigments were purchased from Kremer Pigmente GmbH (Germany): Cobalt Green (PG50), Zinc Iron Yellow (PY119), and Praseodym Yellow (PY159). The organic pigment Isoindoline Yellow (PY139) was obtained from Synthesia (Czech Republic). The chemical compositions of the pigments are presented in Table 1.

### 2.2. Methods

Compounding process was carried out on a Brabender Measuring Mixer N50 (Duisburg, Germany). The composites containing 100 phr (parts per hundred part of rubber) ethylene-norbornene copolymer were filled with 1 phr or 3 phr of a spinel pigment. The EN/spinel pigment composites were prepared at a rotor speed of 50 rpm with a chamber temperature of 110 °C. Afterwards, the EN/pigments composites were molded into films with a thickness of 1 mm using a hydraulic press for 5 min at 110 °C. The thermal properties of the EN/pigments blends were measured using a Mettler Toledo Thermogravimetric Analyzer TGA (Columbus, OH, USA). Composite samples of approximately 10 mg were placed in an aluminum oxide crucible and heated from 25 °C to 600 °C in an argon atmosphere, with a heating rate of 10 °C/min. Microscale combustion calorimetry was used to measure the flammability of the EN/spinel pigment composites. Similar fragments of each composite weighing approximately 2.5 mg were measured on an MCC micro-calorimeter (Fire Testing Technology Limited). Flammability tests were performed with the following parameters: pyrolyzer temperature 750 °C and combustor temperature 900 °C. Mechanical properties were measured using a universal tensile testing machine Zwick/Roell 1435 (Zwick Roell Group, Ulm, Germany) at a uniform crosshead speed of 500 mm/min. Testing was carried out following ISO 37 standard guidelines. Elongation at break (EB) and tensile strength (TS) were calculated as the average of five measurements (the error of measurement was around ± 10%). The aging coefficient K was estimated according to the formula [36]:

(1) $$K=\frac{{\left(\mathrm{TS}\cdot \mathrm{EB}\right)}_{\mathrm{after}\;\mathrm{ageing}}}{{\left(\mathrm{TS}\cdot \mathrm{EB}\right)}_{\mathrm{before}\;\mathrm{ageing}}}$$

Diffuse UV-Vis spectra of the pigment powders were recorded on an Evolution 201/220 UV–Visible Spectrophotometer (Thermo Fisher Scientific, Waltham, MA, USA). The samples were stored under normal conditions and the spectra were recorded in the range of 200–1100 nm, directly in air with no further pretreatment. The accuracy of the apparatus was ±0.8 nm and the repeatability was ≤0.05 nm. Color changes were recorded in the wavelength range of 360–740 nm with a CM-3600d spectrophotometer (Konica Minolta Sensing Inc., Osaka, Japan) and represented in the CIELAB color space. Obtained data were calculated as the average of five measurements from the different places of sample. The measurement error was around ±10%. Total color change ΔE was calculated according to the following equation [37]:

(2) $$\Delta E\;=\sqrt{{\Delta L}^{2}+{\Delta a}^{2}+{\Delta b}^{2}}$$

where ∆L, ∆a, and ∆b represent the differences between the initial and final values for L (brightness), a (red-green color coordinate), and b (yellow-blue color coordinate), respectively.

Infrared absorbance spectra were obtained using a Thermo Scientific Nicolet 6700 (Waltham, MA, USA) for attenuated total reflection Fourier transform infrared spectroscopy (ATR-FTIR). The ATR-FTIR technique was applied in the wavenumber range of 4000–500 cm^−1^ to analyze the formation of the oxidation products. To calculate the carbonyl index (CI), the changes in the relative absorbance intensity of the ketone group A_>C=O_ (corresponding to 1800–1680 cm^−1^) were compared to those of methylene group A_-CH2-_ (at 3000–2800 cm^−1^), according to the formula [38]:

(3) $$CI=\frac{A_{>C=O}}{A_{-CH_{2}-}}$$

The UV aging process was simulated in an Atlas UV 2000 (Ametek Atlas, Linsengericht, Germany) apparatus. Accelerated aging was performed at wavelength λ = 343 nm over 900 h. The procedure combined consecutively repeating segments: a daily segment (radiation intensity 0.7 W/m^2^, temperature 60 °C, duration 8 h) and a night segment (no UV radiation, temperature 50 °C, duration 4 h). The morphology of the EN copolymer samples colored with different two spinel pigments was evaluated based on scanning electron microscopy (SEM). SEM images were taken using a scanning electron microscope (SEM, Zeiss, ULTRA Plus, Oberchoken, Germany) at magnifications of 5000× and 1000×. Prior to SEM observations, liquid nitrogen-fractured surfaces of the composites were carbon sputtered.

## 3. Results

### 3.1. Morphology and Mechanical Properties of EN Composites

**Figure 1:**
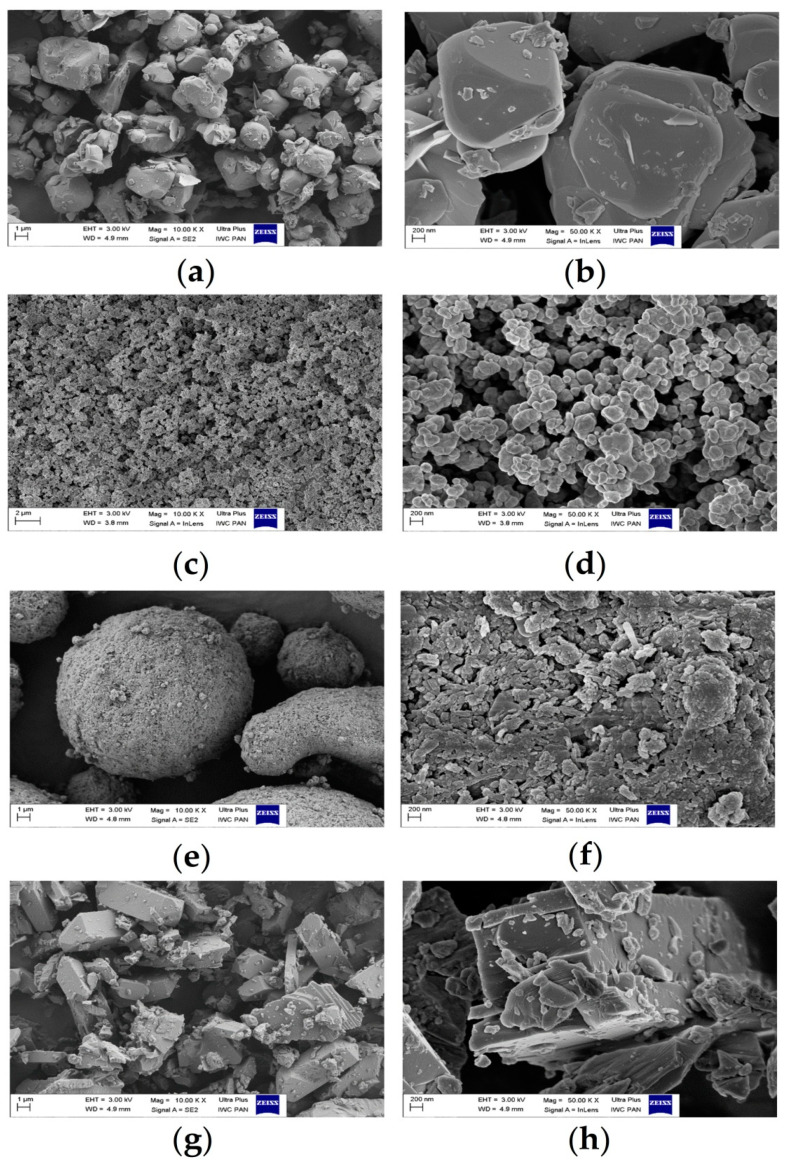
SEM images of the studied spinel pigments at magnification of 10 kx and 50 kx: PG50 (**a**,**b**), PY119 (**c**,**d**), PY139 (**e**,**f**), and PY159 (**g**,**h**).

The ethylene-norbornene (EN) copolymer was compounded with different spinel pigments at 1%wt. The EN films containing spinels exhibited different colors and retained moderate levels of clarity. The morphologies of both the spinel pigments and the EN composites filled with the colorants were analyzed using scanning electron microscopy (SEM) at different magnifications. Figure 1 shows SEM images of the pigment powders. It is known from the literature that the optical properties of a pigment, in particular the color and hiding power, depend on the form and dimensions of its particles [39]. Usually, the particles appear in the form of different conglomerates, in which individual particles of various forms and dimensions can be identified. Based on the microscopic photos, it can be seen that the particles of the spinel pigments differed in shape and size. The PG50 pigment particles exhibited oval cube shapes, the PY119 pigment had tiny particles that were spherical in shape, the PY159 particles were brick-like in shape, whereas the particles of the organic PY139 pigment took the form of large spherical agglomerates. The largest clusters of agglomerates were noted for the PY139 pigment, the lateral dimensions of which were in the order of several dozen microns. Pigment PY119 had the smallest particle size, at about 200 nm.

**Figure 2:**
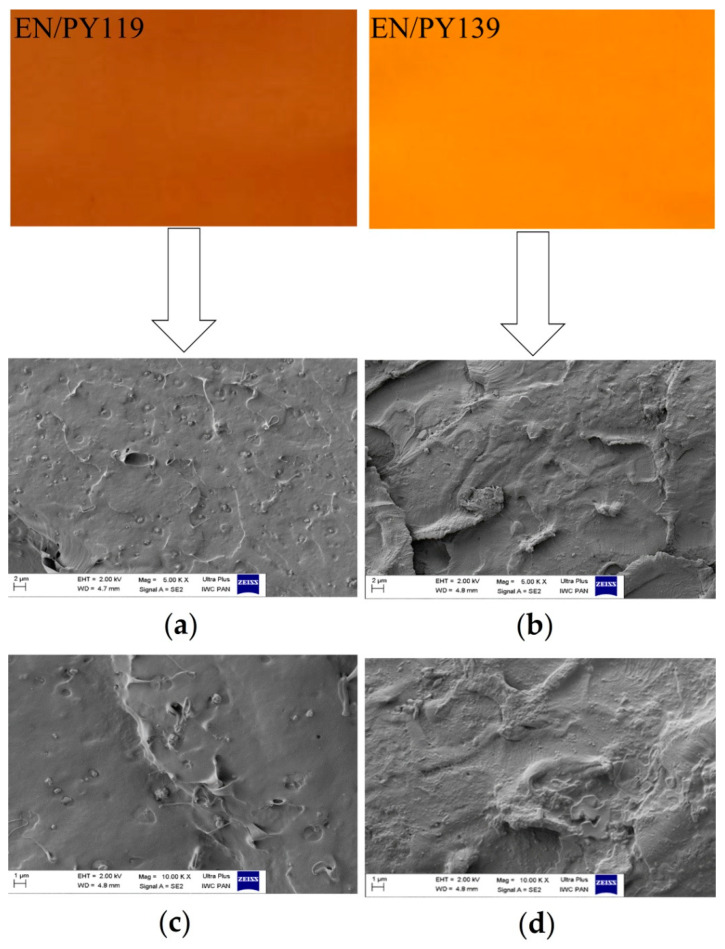
Digital photos and SEM images showing the EN copolymer containing PY119 (**a**,**c**) and PY139 (**b**,**d**) at different magnitudes.

The distribution of pigment particles in the polymer matrix plays an important role in determining its optical properties and can have a decisive impact on the photostability of the polymer composite. Generally, spherically shaped pigment particles exhibit higher hiding power than other particles of a given size. For the purposes of comparison, Figure 2 presents digital photographs and SEM images of the EN composites colored with PY119 and PY139 pigments. In contrast to the PY119 particles, which were easily distinguished in the cross-section of the EN/PY119, the particles of PY139 were not perceptible in the SEM images of the EN polymer. The SEM analysis revealed that the tiny particles of the PY119 spinel pigment exhibited a tendency to accumulate into larger clusters in the polymer matrix. Thus, the relatively small irregular clusters visible in the SEM images of the EN/PY119 samples can be ascribed to agglomerations of particles with different sizes (up to 1 μm). Despite the presence of agglomerates of PY119 particles, the color of the dyed EN was generally uniform.

**Figure 3:**
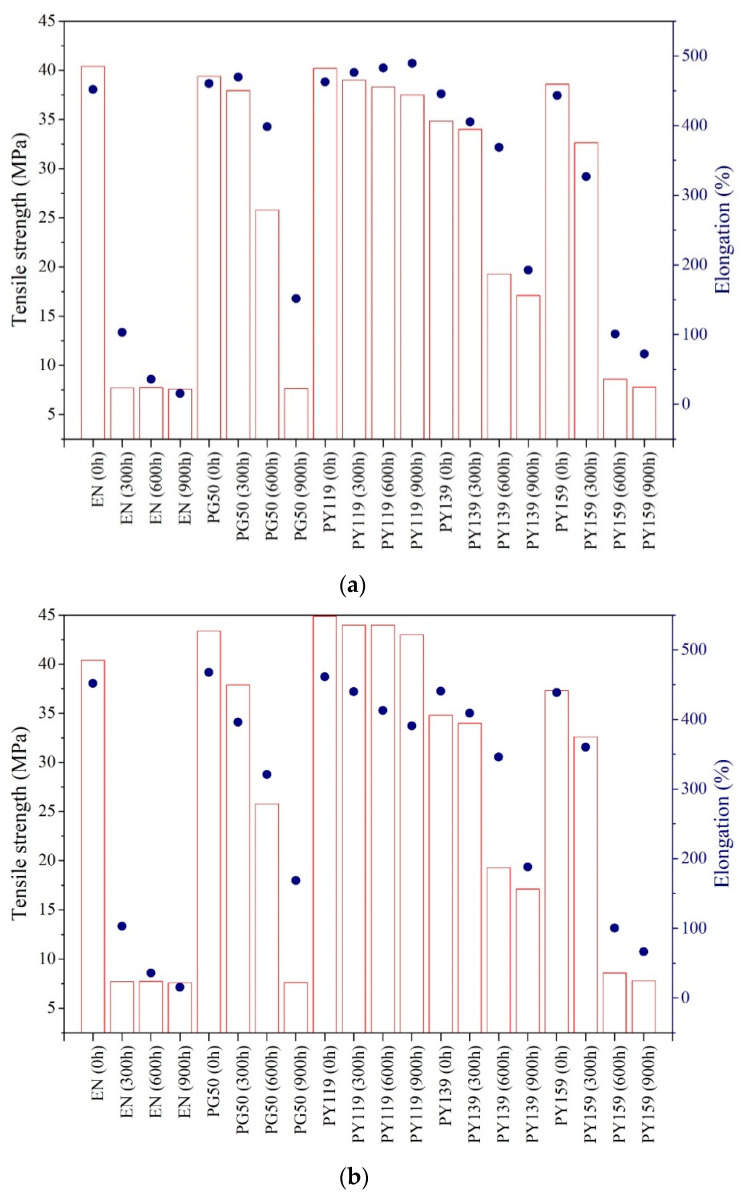
Retention of strength and elongation at break for EN films containing 1 phr (**a**) and 3 phr (**b**) of spinels exposed to 300, 600, and 900 h of accelerated UV aging.

**Figure 4:**
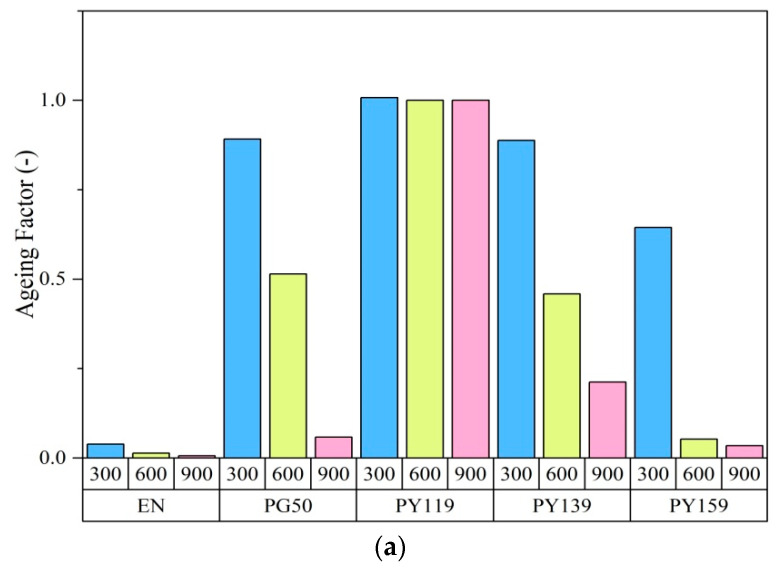

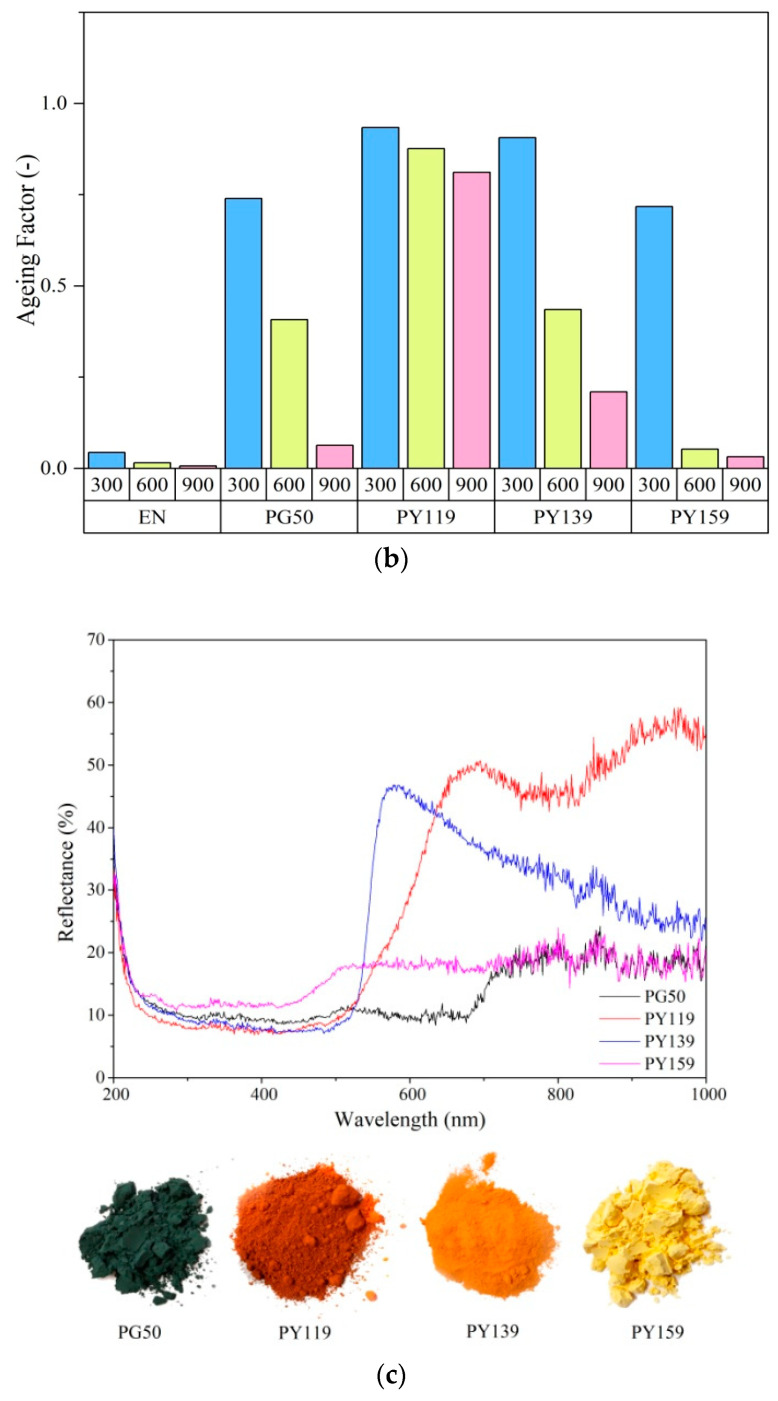
Aging factor of EN as a function of aging time for EN samples containing 1 phr (**a**) and 3 phr (**b**) of pigment; reflectance spectra of the pigment powders (**c**).

Ethylene-norbornene copolymers display interesting useful physico-chemical properties, such as glass-like transparency, rigidity, heat and chemical resistance, and low permeability to gas and water. Due to its good dielectric resistance over a wide temperature range, EN can be used to replace other polymers, for example polypropylene in thin film capacitors. There are numerous studies in the literature describing the synthesis or modification of different grades of EN copolymers, but only a few have investigated the impact of UV aging on these materials [40,41]. It is known that long-term ultraviolet radiation causes photooxidative degradation, which results in the breakage of polymer chains, producing radicals and reducing the molecular weight [42]. Eventually, after a certain time, the mechanical properties of the material deteriorate to the point that it can no longer be used. We therefore studied the strength behavior of the samples following different periods of exposure to UV irradiation. Figure 3 and Figure 4 summarize the results of the mechanical tests performed for the samples subjected to long-term UV radiation.

The application of 1 phr or 3 phr spinel pigments did not produce marked alterations in the mechanical parameters of the EN composites in comparison to the reference sample. The exception was organic pigment PY 139, which at a higher concentration contributed to a decrease in tensile strength of about 6 MPa in comparison to the reference. Before aging, the reference samples of EN exhibited tensile strengths (TS) of 40.6 MPa. However, after 300 h of UV irradiation, degradation of the reference sample led to a decrease in the tensile value by up to 8 MPa. This was most likely caused by the reduction in mobility due to cross-linking and chain scission. The composites containing pigments showed better resistance to UV irritation, as the TS values and aging factors remained almost unchanged after 300 h of photoaging. The samples colored with PG50 and PY119 demonstrated the most minor changes in terms of mechanical properties. After 900 h of aging, only the sample containing the pigment PY 119 showed TS and EB values similar to the sample before aging, at 39 MPa and 500%, respectively. Further aging of the samples with PG 50 and PY159 led to a dramatic reduction in TS (around 7 MPa) and EB (around 40%) in comparison to the unaged samples. The organic colorant PY 139 was found to provide less-efficient protection than the spinel pigments, especially during long-term UV radiation. The sample of EN with PY139 showed TS values of 23 MPa and 19 MPa after 600 h and 900 h aging, respectively. After 900 h, the copolymer containing 1 phr of spinel PY119 showed aging mechanical parameters similar to those of the starting sample. Only in the case of this pigment, its higher concentration in polymer matrix (3 phr) contributed to extend the UV stability of the samples. The progress of degradation was also expressed as an aging factor, which is the combination of tensile strength and elasticity before and after aging (Figure 4a,b). The aging factors of the pure EN composites were much lower than 1 (after 300 h), which confirmed that they had undergone considerable degradation. The durability of the EN films was enhanced by the application of spinels (with concentrations of 1 phr and 3 phr) in comparison to the PY139 organic pigment. The results of reflectance studies (Figure 4c) showed that the pigment powders absorbed UV light and reflected other irradiation in the solar spectra. The samples were subjected to irradiation at a wavelength of 343 nm. The most significant protection against UV aging was provided by PY119, however the level of UV absorption between 200 and 400 nm for PY119 was at similar level like in case of other pigments. From the current study it is not clear, why PY119 provided the most efficient protection against UV aging. Different effect of pigments on the aging stability of polymers may be related to their chemical composition and impact on thermal conductivity of the polymer composite. However, this effect was not confirmed and needs to be further considered in future research.

### 3.2. Colorimetric Study and Carbonyl Index Investigation

**Figure 5:**
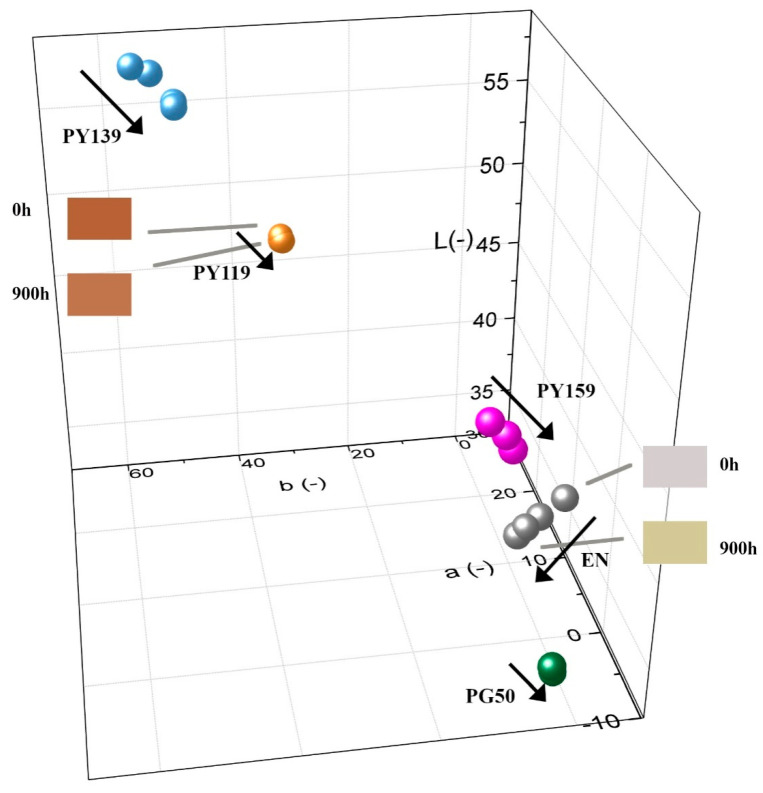
Colorimetric measurements presented as lightness (L), green-red coordinate and blue-yellow coordinate changes for EN and EN/spinel composite samples with concentrations of 1 phr after accelerated UV aging (arrows show the direction of aging time).

Generally, it is known that polymer materials exposed to solar irradiation tend to change their color easily. The CIE-Lab scale provides a standardized photometric system in which color is characterized by three parameters, a, b, and L, and where ΔE is the total change of color. The range of values for lightness (L) was from 0 to 100, where values near to 0 denote black and near to 100 denote white. Spectrophotometer tests provided information about the reflectance curve as a function of wavelengths characteristic for the visible range, and thus numerically described the perceived color of the object. The a (red-green axis) and b (blue-yellow axis) parameters were described as numerical correlates. Changes in hue were plotted as +a red, −a green, and changes in chroma as +b yellow and –b blue in a three-dimensional graph (Figure 5). Color measurements were performed for samples before aging and after 300, 600, or 900 h of UV-accelerated aging. As expected, the sample of EN copolymer changed color rapidly, which indicates that the polymer matrix is not photostable by itself. The values for b increased and the L values decreased, indicating that the EN sample turned yellow and darkened.

**Figure 6:**
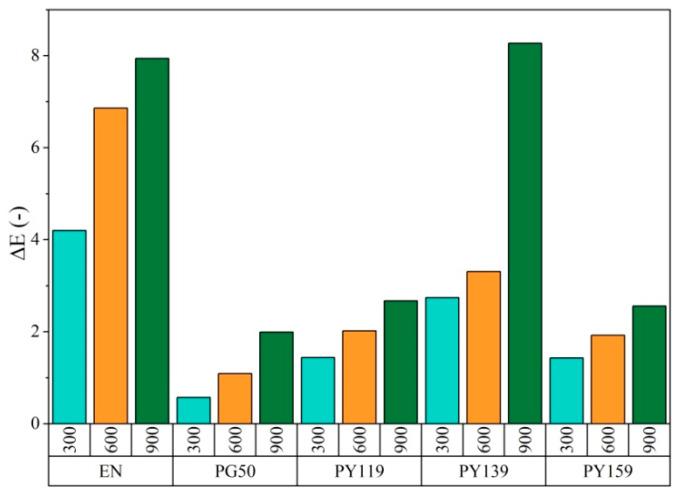
Total color changes as a function of the aging time for EN and EN/pigment samples at concentrations of 1 phr.

Based on the Figure 5, one sees that all the incorporated pigments exhibited appreciable coloring ability. The spinel pigments (PG50, PY119, PY159) showed great photostability in the EN matrix compared to the composite filled with PY139 organic pigment and the EN reference sample. Incorporation of the spinel pigments PG50, PY119, and PY159 increased the photostability of the composites noticeably (Figure 5 and Figure 6). However, after 900 h of UV aging the sample with organic pigment PY139 exhibited comparable changes in ΔE to the reference sample. The poor photostability of this composite may have been due to the different chemical structure of the pigment. At high temperatures and under long-term UV irradiation pigments may undergo several various degradation reactions. These reactions include dehydroxylation, oxidation, changes in the crystal structure, and decomposition. The aging process probably induced structure changes or even decomposition of pigments, or at the least led to unacceptable alterations in color or gloss [43]. Organic PY139 possesses carbonyl groups, which are susceptible to photodegradation. Therefore, the stable inorganic structures of the spinel pigments seem to show better photostability. After 900 h, the reference sample and EN/PY139 showed the highest values for ΔE (above 6), whereas the total color changes for the samples containing the inorganic colorants were around 2. A similar tendency was observed for the EN samples containing 3 phr of the pigments.

**Figure 7:**
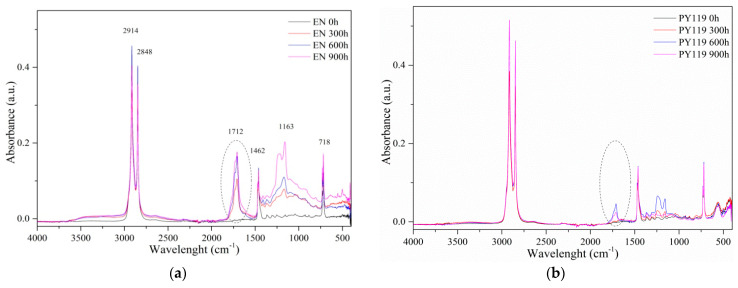

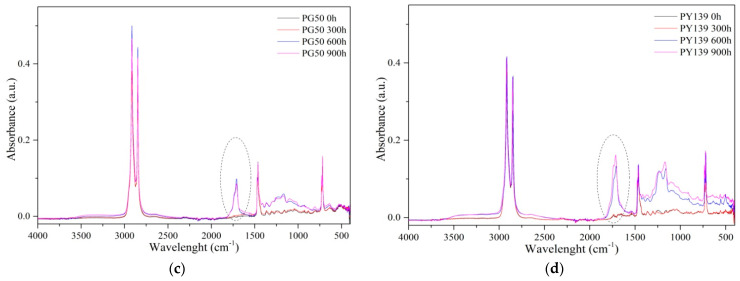
FTIR spectra of the EN copolymer (**a**), EN copolymer with PY119 (**b**), PG50 (**c**), and PY139 (**d**) at different aging times (pigment concentration 1 phr).

Further changes upon UV aging of EN composites was evaluated by Fourier transform infrared spectroscopy analysis. FTIR spectroscopy revealed significant changes in the aging behavior of the EN films. Figure 7 shows the photooxidation profiles of the EN, measured over 900 h of UV irradiation. As expected, exposure to UV irradiation led to structural changes in the composites, which were observed on the absorbance spectra as increases in bands characteristic for carbonyl groups (1600–1800 cm^−1^). The highest increment was noticed for EN at precisely 1712 cm^−1^, which is attributed to the carbonyl groups (C=O). Other characteristic absorbance maximums observed for EN, such as 2915 and 2847 cm^−1^, might be related to the stretching vibration of CH_2_. The peak at 1462 cm^−1^ can be assigned to vibrations of C-O-C, and the band at 718 cm^−1^ is typical for CH_2_ rocking vibrations [44].

**Figure 8:**
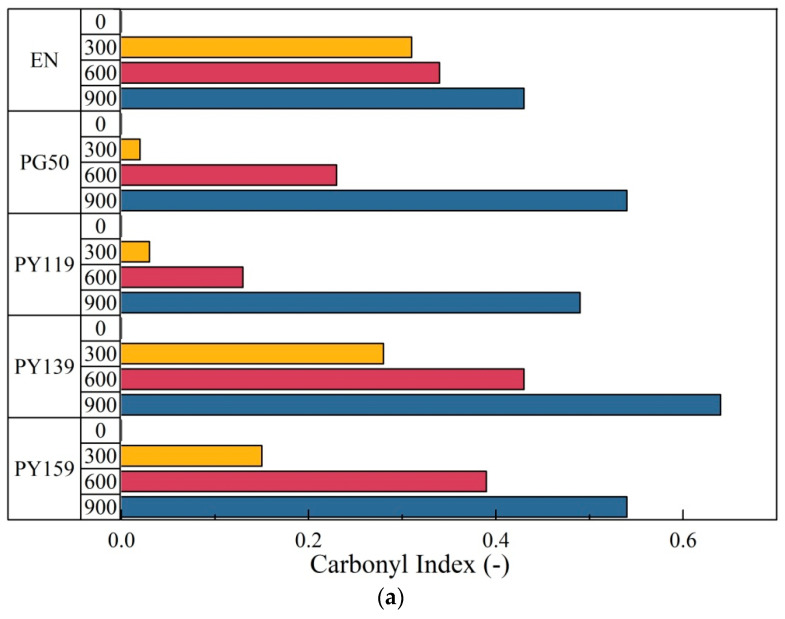

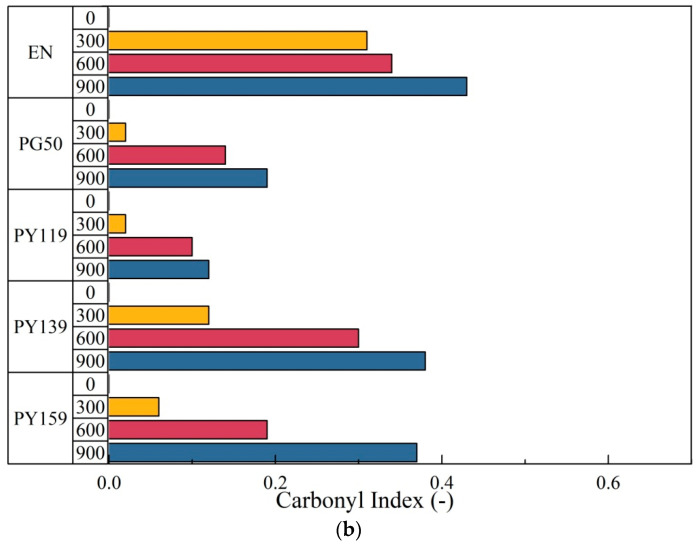
Carbonyl index as a function of the UV irradiation of EN and EN/pigment samples containing 1 phr (**a**) and 3 phr (**b**) of pigment.

The main modifications in the IR spectra of the copolymer films were observed in the carbonyl regions. Therefore, the presence of photooxidized products at 1712 cm^−1^ was reflected in the carbonyl index [38]. We used the intensity of this band as an indicator of the degree of composite degradation. After 300 h of aging, the highest carbonyl index value was observed for the reference sample (Figure 8). The films containing even 1 phr of spinel pigments showed considerably lower concentrations of photoxodized products for the same irradiation time. The highest values after 600 h were observed for the EN and EN colored with organic pigment (PY139) films, indicating pronounced oxidation of the surfaces of the materials. The concentration of photooxidized products, also expressed in terms of the carbonyl index, was found to be the lowest for the sample containing PY 119 (below 0.2).

After 900 h of irradiation, the carbonyl index values increased for all studied samples above 0.5. Pilar et al. [45,46] studied the photooxidation of polypropylene and ethylene-norornene copolymers induced by Xenon light. Higher concentrations of oxidation products were found in the non-stabilized samples of EN copolymer than in the non-stabilized PP. Therefore, it seems important to select pigments capable of enhancing the photostability of EN copolymer and delay surface photooxidation. Based on our results, PY 119 and PG50 are able to protect the surface of EN copolymers against UV irradiation for up to 600 h. The literature describes several different strategies to improve the stability of EN copolymers, including the application of commercial stabilizers such as Irganox 1010 or Tinuvin 770. Pigments PY 119 and PG50 can be considered as complementary additives for use with commercial stabilizers such as phenolic antioxidants or radical scavengers (for example HAS). Higher concentrations of the colorants provided better protection. The carbonyl indexes for the colored samples with 3 phr of pigment were lower than those for the films containing 1 phr. This was most likely related to the higher concentration of the pigment next to the polymer surface.

### 3.3. Dynamic-Mechanical Analysis of EN Composites (DMA)

**Figure 9:**
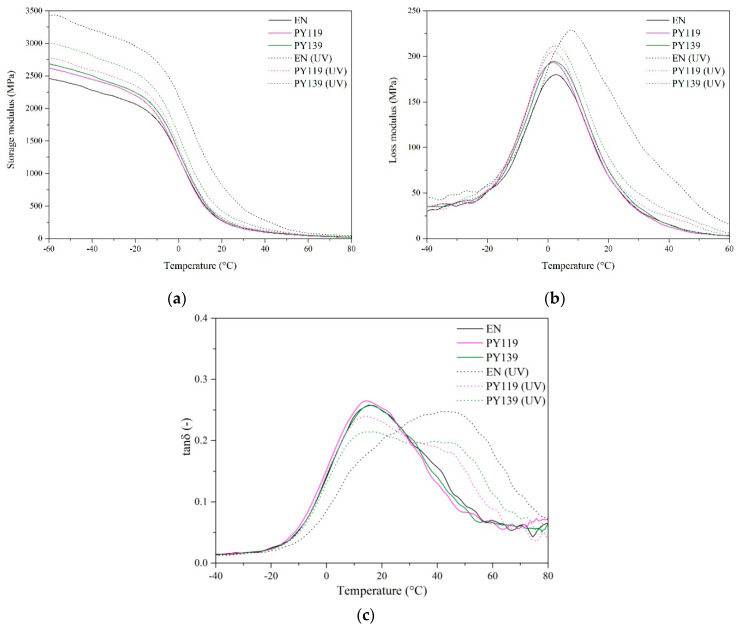
DMA results for EN composites (before and after 900 h UV aging) filled with different pigments: (**a**) storage modulus, (**b**) loss modulus, and (**c**) tanδ versus temperature at concentrations of 1 phr.

Dynamic mechanical analysis (DMA) was performed to investigate the viscoelastic behavior of the EN composites filled with different spinel pigments following long-term UV aging. The alterations in the dynamic mechanical properties of the studied composites were examined at 5 Hz of frequency, in a temperature range from −60 to +60 °C. The storage modulus (E’), loss modulus (E”), and loss tangent (tanδ) over the temperature for all samples before and after 900 h of aging are presented in Figure 9a–c. The Tg values of the EN compounds were calculated from the maxima (peaks) of the tanδ curve (Figure 9c). The results show that the application of 1 phr spinel pigments did not cause any change in Tg, which for all composites was approximately 16 °C. The results for Tg are in line with our previous studies on EN copolymers [47,48].

Based on the DMA results, one sees that the values for the storage modulus of all the studied samples increased after aging. This increase is particularly noticeable in the case of the reference sample (EN), for which the E’ value at 25 °C was as much as 400 MPa higher after aging. This means that the samples were progressively degraded, resulting in greater stiffness and simultaneously lower flexibility, due to the formation of photoproducts and a crosslinked network. A similar effect had been observed previously for polymer composites exposed to long-term aging [49]. Interestingly, after aging the loss modulus peak of the unprotected EN sample rose significantly and shifted toward higher temperatures, which further confirms that the EN copolymer was strongly degraded due to UV exposure. The composites with pigment PY119 showed higher E” peaks, but the increases were much lower than for the unprotected sample or the sample with organic PY139, indicating better resistance to UV radiation after the incorporation of the spinel pigments. The progressive aging of the studied samples was also reflected in the visible changes in the shapes of their tanδ peaks, which became considerably wider compared to the unaged materials. A significant increase in Tg (from 16 up to about 44 °C) was also observed in the case of the aged unfilled EN copolymer, most likely due to the complex changes that occurred in the polymer structure.

### 3.4. Thermal Properties and Combustion Behavior of EN Composites

**Figure 10:**
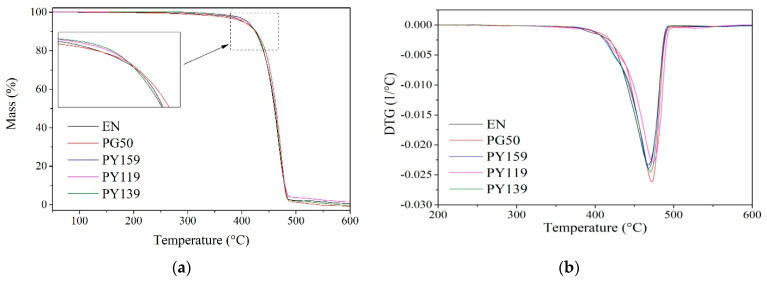
Thermogravimetric curves of the EN/pigment samples: (**a**) TGA, (**b**) DTG.

The incorporation of the spinel pigments into the EN copolymer had ambiguous effects on the thermal stability of the resulting composites. The addition of 1 phr of organic pigment (PY139) to the EN composites resulted in a slight increase in the T5 parameter. It can be assumed that the carbon ring structure of the PY139 pigment improved carbonization processes. The carbonized boundary layer inhibits diffusion of thermal degradation products in the gas phase (Table 2, Figure 10). The inorganic cobalt pigment (PG50) also had a positive effect on the T5 parameter. Generally, the initiation of thermal transformations of polymers occurs as a result of the decomposition of hydroperoxide groups in the polymer macromolecules, as well as of those formed during heating in an air atmosphere. The presence of a metal of variable valence in the pigment structure (e.g., PY119) caused the hydroperoxide groups in the EN to break down into free radicals and hydroxyl anions (homogeneous–heterogeneous decomposition). The macroradicals resulting from the splitting of hydroperoxide groups reacted with each other or initiated proton cleavage, including the chains of other polymer macromolecules (thermal crosslinking reactions) [50,51]. Thermal cross-linking reactions are accompanied by cyclization as well as degradation of polymer chains (the presence of radicals and hydroxyl ions). The predominance of cyclization and thermal cross-linking reactions over degradation reactions is confirmed by the increase in the residue after thermal decomposition (Table 2).

Due to the fact that the presence of 1 phr of the pigments had only slight impact on the thermal stability of the EN copolymer, we decided to perform microcombustion calorimetric test for samples with 3 phr of the pigments. The spinel additives had a positive effect on the flammability of the EN copolymer. Based on the MCC results, the addition of 3 phr of PY139 pigment improved the flame retardancy of the EN copolymer, as evidenced by the significant reduction in HRR and THR parameters, by 15% and 40% compared to the neat copolymer, respectively (Table 3). Given the very small amounts of each sample (several mg) used in the MCC analysis, the reduction in the flammability of the EN/PY139 composite can be assumed to be directly related to the high thermal stability of the pigment, which delayed the thermal decomposition of the composite. Another important factor that should be mentioned when considering the reduced flammability of the EN/PY139 composite is the high content of amino groups in the pigment structure. The nitrogen compounds formed during thermal decomposition of PY139 dilute the combustible destructs of the composite, inhibiting the combustion process. The application of 3 phr of PY119, PY159, or PG50 of the spinel pigments also reduced the HRR and THR parameters, indicating lower flammability. The flame-retardant effects of the PY119 and PY159 inorganic pigments were a direct result of the presence of metal oxides, mainly iron and zinc. Metal oxides can act as radical scavengers, which interrupt high-energy reactions in the gas phase and thus reducing the transfer of heat to the composite boundary layer. Such thermal cross-linking reactions also have an important role in reducing the flammability of the composite with PG50 pigment.

## 4. Conclusions

We have proposed a strategy for improving the photostability of ethylene-norbornene composites by the application of colorful spinel pigments. Three different spinel pigments were applied as colorants in ethylene-norbornene (EN) copolymer: Cobalt Green, Zinc Iron Yellow, and Praseodym Yellow. An EN composite with the addition of the organic pigment PY 139 was prepared for the purpose of comparison. The colorful EN composites with spinel pigments were subjected to long-term UV aging (300, 600, and 900 h), as well as thermal, mechanical, and combustion tests. Color changes were measured in the CIE Lab color space. The results confirmed that the spinel pigments were effective in protecting the EN copolymer against UV radiation. This contrasts with the pure copolymer, which showed a dramatic decrease in tensile strength from 40 MPa up to 8 MPa after 900 h of aging. Dynamic mechanical analysis revealed a significant increase in the storage and loss of moduli of the pure EN composite after UV aging, which indicates an increase in the stiffness of the composite because of progressive photodegradation. The organic pigment protected the polymer matrix against UV exposure, but was markedly less effective than the spinel pigments. This was evidenced by the higher total color change (ΔE) and carbonyl index (CI) determined for the EN/P139 composite compared to the film containing especially PY 119 pigment. The flammability of the EN copolymer also reduced considerably following the incorporation of spinel pigments. Based on microcombustion analysis, the application of 3 phr of the PG50, PY119 or PY159 pigments reduced the heat release rate parameter from 1857 W/g (for neat EN composite) to 1433 W/g, 1754 W/g, and 1480 W/g, respectively. This effect can be explained by the presence of metal oxides in the spinel pigments, which act as radical scavengers reducing heat transfer to the boundary layer of the composite. Overall, this study shows that spinel pigments can improve the resistance of polymer composites to elevated temperatures and long-term exposure to UV radiation, while providing aesthetic properties to the final products.

## Figures and Tables

**Table 1:** Chemical formulas of pigments.

Name	Abbreviation	Formula
Cobalt Green	PG50	Co,Ni,Ti,Zn
Zinc Iron Yellow	PY119	Fe_2_O_3_.ZnO
Praseodym Yellow	PY159	(Zr,Pr)SiO_4_
Isoindoline Yellow	PY139	C_16_H_9_N_5_O_6_

**Table 2:** Thermal stability of EN/pigment samples at concentrations of 1 phr.

Sample Code	T_5_ (°C)	T_10_ (°C)	T_50_ (°C)	Char Residue (%)
EN	403	424	460	0.58
PG50	405	426	464	1.12
PY119	410	425	462	1.64
PY139	412	425	462	1.00
PY159	409	425	461	1.24

T_5,10,50_—degradation temperatures of 5, 10, and 50% weight of sample loss.

**Table 3:** Combustion parameters of EN/pigment samples at concentrations of 3 phr.

Sample Code	HRR (W/g)	THRR (°C)	THR (kJ/g)	HRC (J/gK)
EN	1857 ± 85	497 ± 5	79.4 ± 5	1851 ± 90
PG50 (3%)	1433 ± 59	496 ± 5	49.9 ± 4	1390 ± 63
PY119 (3%)	1754 ± 72	496 ± 5	54.9 ± 4	1698 ± 62
PY139 (3%)	1584 ± 64	487 ± 5	47.9 ± 3	1535 ± 59
PY159 (3%)	1748 ± 70	494 ± 5	57.0 ± 4	1692 ± 60

HRR—heat release rate; THRR—total heat release rate; THR—total heat release; HRC—heat release capacity.

## Data Availability

Data sharing not applicable.

## References

[1] Fleischmann C., Lievenbrück M., Ritter H. (2015). Polymers and Dyes: Developments and Applications. Polymers.

[2] Zhao X., Meng Q., Liu J., Li Q. (2014). Hydrophobic Dye/Polymer Composite Colorants Synthesized by Miniemulsion Solvent Evaporation Technique. Dye. Pigment..

[3] Marzec A., Szadkowski B., Rogowski J., Maniukiewicz W., Kozanecki M., Moszyński D., Zaborski M. (2019). Characterization and properties of new color-tunable hybrid pigments based on layered double hydroxides (LDH) and 1,2-dihydroxyanthraquinone dye. J. Ind. Eng. Chem..

[4] Guo Y., Ruan K., Shi X., Yang X., Gu J. (2020). Factors Affecting Thermal Conductivities of the Polymers and Polymer Composites: A Review. Compos. Sci. Technol..

[5] Marturano V., Cerruti P., Ambrogi V. (2019). Polymer Additives. Phys. Sci. Rev..

[6] Kiernan J.A. (2001). Classification and Naming of Dyes, Stains and Fluorochromes. Biotech. Histochem..

[7] Harper C. (2006). Handbook of Plastics Technologies: The Complete Guide to Properties and Performance.

[8] Buvaneswari G., Aswathy V., Rajakumari R. (2015). Comparison of Color and Optical Absorbance Properties of Divalent Ion Substituted Cu and Zn Aluminate Spinel Oxides Synthesized by Combustion Method towards Pigment Application. Dye. Pigment..

[9] De Souza L.K.C., Zamian J.R., da Rocha Filho G.N., Soledade L.E.B., dos Santos I.M.G., Souza A.G., Scheller T., Angélica R.S., da Costa C.E.F. (2009). Blue Pigments Based on CoxZn1-XAl_2_O_4_ Spinels Synthesized by the Polymeric Precursor Method. Dye. Pigment..

[10] Jose S., Joshy D., Narendranath S.B., Periyat P. (2019). Recent Advances in Infrared Reflective Inorganic Pigments. Sol. Energy Mater. Sol. Cells.

[11] Molinari C., Conte S., Zanelli C., Ardit M., Cruciani G., Dondi M. (2020). Ceramic Pigments and Dyes beyond the Inkjet Revolution: From Technological Requirements to Constraints in Colorant Design. Ceram. Int..

[12] Llusar M., Zielinska A., Tena M.A., Badenes J.A., Monrós G. (2010). Blue-Violet Ceramic Pigments Based on Co and Mg Co2-XMgxP2O7 Diphosphates. J. Eur. Ceram. Soc..

[13] Zhang L., Deng Z., Liang L., Zhang Y., Meng Q., Wang J., Santamouris M. (2019). Thermal behavior of a vertical greenfacade and its impact on the indoor and outdoor thermal environment. Energy Build..

[14] Gaudon M., Robertson L.C., Lataste E., Duttine M., Ménétrier M., Demourgues A. (2014). Cobalt and Nickel Aluminate Spinels: Blue and Cyan Pigments. Ceram. Int..

[15] Ragupathi C., Vijaya J.J., Kennedy L.J., Bououdina M. (2014). Combustion Synthesis, Structure, Magnetic and Optical Properties of Cobalt Aluminate Spinel Nanocrystals. Ceram. Int..

[16] Shetty K., Renuka L., Nagaswarupa H.P., Nagabhushana H., Anantharaju K.S., Rangappa D., Prashantha S.C., Ashwini K. (2017). A Comparative Study on CuFe_2_O_4_, ZnFe_2_O_4_ and NiFe_2_O_4_: Morphology, Impedance and Photocatalytic Studies. Mater. Today Proc..

[17] Meenakshi P., Selvaraj M. (2018). Bismuth titanate as an infrared reflective pigment for cool roof coating. Sol. Energy Mater. Sol. C.

[18] Escobedo J.F., Gomes E.N., Oliveira A.P., Soares J. (2011). Ratios of UV, PAR and NIR Components to Global Solar Radiation Measured at Botucatu Site in Brazil. Renew. Energy.

[19] Escobedo J.F., Gomes E.N., Oliveira A.P., Soares J. (2009). Modeling hourly and daily fractions of UV, PAR and NIR to global solar radiation under various sky conditions at Botucatu, Brazil. Appl. Energy.

[20] Smith A.E., Comstock M.C., Subramanian M.A. (2016). Spectral Properties of the UV Absorbing and Near-IR Reflecting Blue Pigment, YIn1-XMnxO3. Dye. Pigment..

[21] Mesíková Ž., Trojan M., Šulcovǎ P. (2005). Conditions of Synthesis of Co-Zn-Ti-Cr Spinel Pigment. Ceramics Silikaty.

[22] Llusar M., Forés A., Badenes J.A., Calbo J., Tena M.A., Monrós G. (2001). Colour Analysis of Some Cobalt-Based Blue Pigments. J. Eur. Ceram. Soc..

[23] Liu L., Han A., Ye M., Feng W. (2015). The Evaluation of Thermal Performance of Cool Coatings Colored with High Near-Infrared Reflective Nano-Brown Inorganic Pigments: Magnesium Doped ZnFe_2_O_4_ Compounds. Sol. Energy.

[24] Anand G.T., Kennedy L.J., Vijaya J.J. (2013). Microwave combustion synthesis, structural, optical and magnetic properties of Zn1-xCoxAl_2_O_4_ (0_x_0.5) spinel nanostructures. J Alloys Compd..

[25] Cannio M., Bondioli F. (2012). Mechanical Activation of Raw Materials in the Synthesis of Fe_2_O_3_-ZrSiO_4_ Inclusion Pigment. J. Eur. Ceram. Soc..

[26] Kalendová A. (2000). Application of spinel pigments in anticorrosive heat-resistant coatings. Pigment. Resin Technol..

[27] Anees P., Sreejith S., Ajayaghosh A. (2014). Self-Assembled near-Infrared Dye Nanoparticles as a Selective Protein Sensor by Activation of a Dormant Fluorophore. J. Am. Chem. Soc..

[28] Moura J.C.V.P., Oliveira-Camposa A.M.F., Griffiths J. (1997). The effect of additives on the photostability of dyed polymers. Dye. Pigment..

[29] Klemchuk P.P. (1982). Influance of pigments on the light stability of polymers: A critical review. Polym. Photochem..

[30] Uzelmeier C. (1970). How heat and light affect pigmented polypropylene. SPE J..

[31] Mlinac M., Rolich J., Bravar M. (1976). Photodegradation of colored polyethylene films. J. Polym. Sci. C Polym. Symp..

[32] Steinlin F., Saar W. (1980). Influence of pigments on the degradation of polypropylene fibers on exposure to light and weather. Melliand Textilberichte.

[33] Gilroy H.M., Chan M.G. (1981). Effect of pigments on the aging characteristics of polyolefins. Org. Coat. Appl. Polym. Sci. Proc..

[34] Wijdekop M., Arnold J.C., Evans M., John V., Lloyd A. (2005). Monitoring with reflectance spectroscopy the color change of PVC plastisol coated strip steel due to weathering. Mater. Sci. Tech..

[35] Szadkowski B., Kuśmierek M., Rybiński P., Zukowski W., Marzec A. (2020). Application of Earth Pigments in Cycloolefin Copolymer: Protection against Combustion and Accelerated Aging in the Full Sunlight Spectrum. Materials.

[36] Maciejewska M., Sowinska A., Kucharska J. (2019). Organic Zinc Salts as Pro-Ecological Activators for Sulfur Vulcanization of Styrene-Butadiene Rubber. Polymers.

[37] Maslowski M., Aleksieiev A., Miedzianowska J., Strzelec K. (2021). Common Nettle (*Urtica dioica* L.) as an Active Filler of Natural Rubber Biocomposites. Materials.

[38] Plota A., Masek A. (2021). Plant-Origin Stabilizer as an Alternative of Natural Additive to Polymers Used in Packaging Materials. Int. J. Mol. Sci..

[39] Gueli A.M., Bonfiglio G., Pasquale S., Troja S.O. (2017). Effect of particle size on pigments colour. Color Res. Appl..

[40] Lago W.S.R., Aymes-Chodur C., Ahoussou A.P., Yagoubi N. (2017). Physico-chemical ageing of ethylene–norbornene copolymers: A review. J. Mater. Sci..

[41] Lamonte R., Mac Nally D. (2001). Cyclo olefin copolymers. Adv. Mater. Proces..

[42] Kamweru P.K., Ndiritu F.G., Kinyanjui T.K., Muthui Z.W., Ngumbu R.G., Odhiambo P.M. (2012). Study of Humidity and Uv Wavelength Effects on Degradation of Photo-Irradiated Polyethylene Films Using DMA. J. Macromol. Sci. Part B Phys..

[43] Ghelardi E., Degano I., Colombini M., Mazurek J., Schilling M., Khanjian H., Learner T. (2015). A multi-analytical study on the photochemical degradation of synthetic organic pigments. Dye. Pigm..

[44] Masek A., Plota A. (2021). Influence of a Natural Plant Antioxidant on the Ageing Process of Ethylene-norbornene Copolymer (Topas). Int. J. Mol. Sci..

[45] Pilař J., Michálková D., Šeděnková I., Pfleger J., Pospíšil J. (2011). NOR and nitroxide-based HAS in accelerated photooxidation of carbon-chain polymers; comparison with secondary HAS: An ESRI and ATR FTIR study. Polym. Degrad. Stab..

[46] Pilař J., Michálková D., Šlouf M., Vacková T., Dybal J. (2014). Heterogeneity of accelerated photooxidation in commodity polymers stabilized by HAS: ESRI, IR, and MH study. Polym. Degrad. Stab..

[47] Marzec A., Szadkowski B., Kuśmierek M., Rogowski J., Maniukiewicz W., Rybiński P., Zaborski M. (2020). Impact of Organic-Inorganic Color Additive on the Properties of Ethylene-Norbornene Copolymer. Polym. Test..

[48] Marzec A., Szadkowski B. (2019). Improved Aging Stability of Ethylene-Norbornene Composites Filled with Lawsone-Based Hybrid Pigment. Polymers.

[49] Bhowmick A.K., White J.R. (2002). Thermal, UV- and Sunlight Ageing of Thermoplastic Elastomeric Natural Rubber-Polyethylene Blends. J. Mater. Sci..

[50] Wen X., Wang Y., Gong J., Liu J., Tian N., Wang Y., Jiang Z., Qiu J., Tang T. (2012). Thermal and flammability properties of polypropylene/carbon black nanocomposites. Polym. Degrad. Stab..

[51] Rybiński P., Anyszka R., Imiela M., Siciński M., Gozdek T. (2017). Effect of modified Graphene and carbon nanotubes on the thermal properties and flammability of elastomeric materials. J. Therm. Anal. Calorim..

